# International Brazilian Journal of Urology is among the Top 10 urologic journals - the new Impact Factor is 3.7!

**DOI:** 10.1590/S1677-5538.IBJU.2023.05.01

**Published:** 2023-08-22

**Authors:** Luciano A. Favorito

**Affiliations:** 1 Universidade do Estado do Rio de Janeiro Unidade de Pesquisa Urogenital Rio de Janeiro RJ Brasil Unidade de Pesquisa Urogenital - Universidade do Estado do Rio de Janeiro - Uerj, Rio de Janeiro, RJ, Brasil;; 2 Hospital Federal da Lagoa Rio de Janeiro RJ Brasil Serviço de Urologia, Hospital Federal da Lagoa, Rio de Janeiro, RJ, Brasil

The September-October number of *Int Braz J Urol* is the 24th under my supervision and is very special. In July 23 the Web of Science released shows the 2022 impact factor and the Int Braz J Urol head the impact of 3.7 – the biggest in its history! A great reward for the hard work by the editorial team. With the new impact factor, the Int Braz J Urol today is one of the top 10 urologic journals (Figure-1), an unimaginable feat in 2019 when I took over as editor in chief. We will continue working for another 4 years to reach the top 5 of urology journals. In the cover of this edition, we can see the new impact factor of the International Brazilian Journal of Urology.

**Figure 1:**
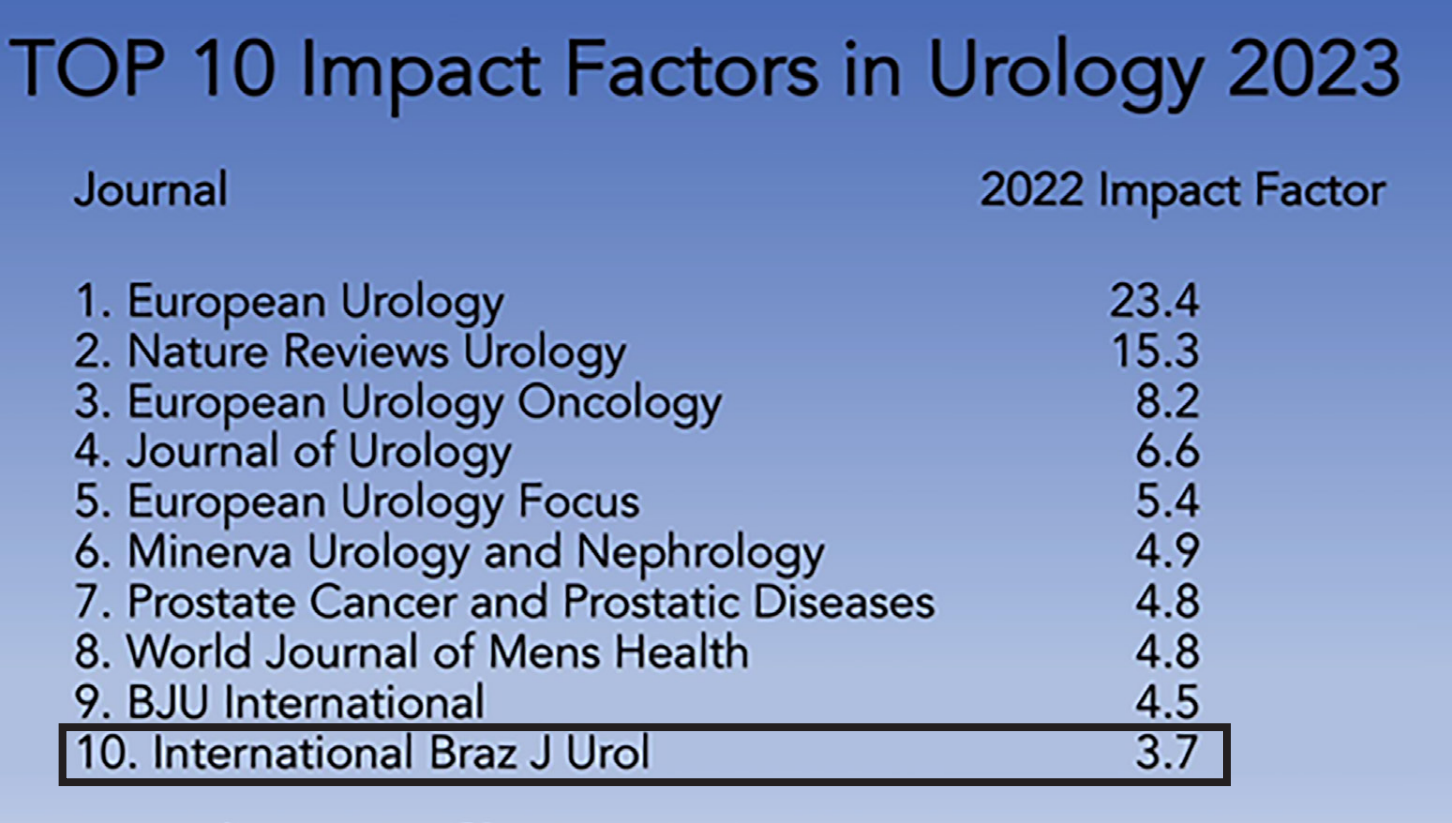
The top 10 impact factors in urology in 2023. International Brazilian Journal of Urology for the first time in his history is in the Top 10.

In this number the Int Braz J Urol presents original contributions with a lot of interesting papers in different fields: Robotic Surgery, Overactive bladder, Endometriosis, Penile Cancer, Enuresis, Urolithiasis, BPH, Ureteroplasty and Varicocele. The papers came from many different countries such as Brazil, Italy, Russia, USA and China, and as usual the editor's comment highlights some of them. The editor in chief would like to highlight the following works:

Dr. He and collegues from China, presented in page 535 (1) a nice meta-analysis about the efficacy and safety of approved oral therapies for overactive bladder and concluded that solifenacin showed better efficacy. For safety, most anticholinergic drugs were more likely to cause dry mouth and constipation, lower doses were better tolerated. The choice of drugs should be tailored to the patient's specific situation to find the best balance between efficacy and safety.

Dr. Diniz and collegues from the Urogenital Research Unit – Rio de Janeiro - Brazil, presented in page 564 (2) a important review about the Urological knowledge and tools applied to diagnosis and surgery in deep infiltrating endometriosis (DIE) and concluded that DIE in the urinary system is common, however the number of publications with high level of evidence is limited. The initial tools for diagnosis are ultrasonography and cystoscopy, but magnetic resonance is the most reliable tool. When the patient has voiding symptoms, the urodynamic examination is crucial. Laparoscopy improves lesion detection and anatomical understanding. This approach must be carried out by professionals with high expertise, since the surgery goes beyond the resection of lesions and includes the preservation of nerve structures and urinary tract reconstruction techniques.

Dr. Tobias-Machado and collegues from Brazil performed in page 580 (3) a nice study about the long-term oncological and surgical outcomes after Video Endoscopic Inguinal Lymphadenectomy (VEIL) in patients with penile cancer and concluded that VEIL seems to offer appropriate long term oncological control with minimal morbidity. In the absence of non-invasive stratification measures such as dynamic sentinel node biopsy, VEIL emerged as the alternative for the management of non- bulky lymph nodes in penile cancer.

Dr. Dahan and collegues form Brazil performed on page 590 (4) an interesting study about the asthma treatment in patients with enuresis: and the repercussions on urinary symptoms and concluded that controlling asthma in children with primary enuresis resulted in a significant increase in dry nights.

Dr. Xie and collegues form China performed on page 599 (5) a nice study about a nomogram to predict the risk of adverse outcomes in patients with residual stones following percutaneous nephrolitothomy (PCNL) and concluded that larger diameter of residual stones, positive urine culture, and previous stone surgery were significant predictors associated with adverse outcomes in patients with residual stones after PCNL. This nomogram could help to assess the risk of adverse outcomes quickly and effectively in patients with residual stones after PCNL

Dr. Maida and collegues from Italy performed on page 608 (6) a nice study about the predictors of early catheter replacement after HoLEP and the results from a high-volume laser center and concluded that the presence of indwelling urinary catheter before surgery, bladder wall modifications and the maintenance of anticoagulants/antiplatelets therapy were shown to be independent predictors of early catheter replacement after HoLEP (Holmium Laser Enucleation of the Prostate).

Dr. Guliev and collegues from Russia performed on page 619 (7) an nice study about the laparoscopic ventral onlay ureteroplasty with buccal mucosa graft for complex proximal ureteral stricture and concluded that patients with proximal ureteral strictures could be effectively treated by laparoscopic ventral onlay ureteroplasty with a buccal mucosa graft.

The Editor-in-chief expects everyone to enjoy reading.

## References

[1] He W, Huang G, Cui W, Tian Y, Sun Q, Zhao X (2023). Comparative assessment of efficacy and safety of approved oral therapies for overactive bladder: a systematic review and network meta-analysis. Int Braz J Urol.

[2] Diniz ALL, Resende JAD, de Andrade CM, Brandão AC, Gasparoni MP, Favorito LA (2023). Urological knowledge and tools applied to diagnosis and surgery in deep infiltrating endometriosis - a narrative review. Int Braz J Urol.

[3] Tobias-Machado M, Ornellas AA, Hidaka AK, Medina LG, Mattos PAL, Besio RS (2023). Long-term oncological and surgical outcomes after Video Endoscopic Inguinal Lymphadenectomy (VEIL) in patients with penile cancer. Int Braz J Urol.

[4] Dahan P, de Oliveira PMN, Brum AR, Ribeiro ACP, Figueiredo AA, de Bessa J, Bastos JM (2023). Treating asthma in patients with enuresis: repercussions on urinary symptoms. Int Braz J Urol.

[5] Xie F, Deng S, Fei K, Xu H, Zhang H (2023). Nomogram to predict the risk of adverse outcomes in patients with residual stones following percutaneous nephrolithotomy. Int Braz J Urol.

[6] Di Maida F, Cadenar A, Grosso AA, Lambertini L, Giudici S, Paganelli D, Salamone V, Mari A, Salvi M, Minervini A, Tuccio A (2023). Predictors of early catheter replacement after HoLEP. Results from a high-volume laser center. Int Braz J Urol.

[7] Guliev BG, Komyakov B, Avazkhanov Z, Shevnin M, Talyshinskii A (2023). Laparoscopic ventral onlay ureteroplasty with buccal mucosa graft for complex proximal ureteral stricture. Int Braz J Urol.

